# Rapid Detection of MCR-Mediated Colistin Resistance in Escherichia coli

**DOI:** 10.1128/spectrum.00920-22

**Published:** 2022-05-26

**Authors:** Haijie Zhang, Feiyu Yu, Xiaoyu Lu, Yan Li, Daxin Peng, Zhiqiang Wang, Yuan Liu

**Affiliations:** a College of Veterinary Medicine, Yangzhou University, Yangzhou, China; b Jiangsu Co-innovation Center for Prevention and Control of Important Animal Infectious Diseases and Zoonoses, Yangzhou University, Yangzhou, China; c Institute of Comparative Medicine, Yangzhou University, Yangzhou, China; d Joint International Research Laboratory of Agriculture and Agri-Product Safety, the Ministry of Education of China, Yangzhou University, Yangzhou, China; Forschungszentrum Jülich GmbH

**Keywords:** antibiotic resistance, *mcr-1*, colistin, mRNA biomarker, antibiotic susceptibility determination

## Abstract

Colistin is one of the last-resort antibiotics for infections caused by multidrug-resistant Gram-negative bacteria. However, the wide spread of novel plasmid-carrying colistin resistance genes *mcr-1* and its variants substantially compromise colistin's therapeutic effectiveness and pose a severe danger to public health. To detect colistin-resistant microorganisms induced by *mcr* genes, rapid and reliable antibiotic susceptibility testing (AST) is imminently needed. In this study, we identified an RNA-based AST (RBAST) to discriminate between colistin-susceptible and *mcr-1*-mediated colistin-resistant bacteria. After short-time colistin treatment, RBAST can detect differentially expressed RNA biomarkers in bacteria. Those candidate mRNA biomarkers were successfully verified within colistin exposure temporal shifts, concentration shifts, and other *mcr-1* variants. Furthermore, a group of clinical strains were effectively distinguished by using the RBAST approach during the 3-h test duration with over 93% accuracy. Taken together, our findings imply that certain mRNA transcripts produced in response to colistin treatment might be useful indicators for the development of fast AST for *mcr*-positive bacteria.

**IMPORTANCE** The emergence and prevalence of *mcr-1* and its variants in humans, animals, and the environment pose a global public health threat. There is a pressing urgency to develop rapid and accurate methods to identify MCR-positive colistin-resistant bacteria in the clinical samples, providing a basis for subsequent effective antibiotic treatment. Using the specific mRNA signatures, we develop an RNA-based antibiotic susceptibility testing (RBAST) for effectively distinguishing colistin-susceptible and *mcr-1*-mediated colistin-resistant strains. Meanwhile, the detection efficiency of these RNA biomarkers was evidenced in other *mcr* variants-carrying strains. By comparing with the traditional AST method, the RBAST method was verified to successfully characterize a set of clinical isolates during 3 h assay time with over 93% accuracy. Our study provides a feasible method for the rapid detection of colistin-resistant strains in clinical practice.

## INTRODUCTION

Antibiotics have saved thousands of lives in recent decades. However, antibiotic resistance has been rising as a result of the overuse and abuse of antibiotics in clinical, agricultural, or other settings (1). If not controlled, antimicrobial resistance (AMR) will cost the world economy more than 210 trillion dollars, with 10 million people dying each year from AMR infections (https://amr-review.org/). Colistin, a member of cationic polypeptide antibiotics, is considered as one of the final effective therapeutic options for carbapenems-resistant Enterobacteriaceae (CRE) infected patients (2, 3). The emergence and prevalence of bacterial resistance to this antibiotic have been increasing rapidly due to the wide use of colistin in animal feeding and plant agriculture, as well as human medicine. Since the first *mcr-1* gene located in the plasmid was identified in a pig-source E. coli in 2015 (4), *mcr-1*-conferred colistin resistance in Enterobacteriaceae has been documented in humans, animals, and the environment all over the world. MCR-1 belongs to the phosphoethanolamine (PEA) transferase enzyme family that can add PEA to lipid A when expressed in E. coli (5). Given the fact that the antibacterial mechanism of colistin is based on electrostatic interaction between its amino groups and lipid A subunits of lipopolysaccharide (LPS), MCR expression reduces LPS's net charge, resulting in detectable colistin resistance (6, 7). To present, 10 different *mcr-1* variants (*mcr-1*–*mcr-10*) have been identified in bacteria isolated from humans, animals, foods, and the environment (8). Since the first discovery of the coexistence of extended-spectrum beta-lactamase (ESBL) and *mcr-1* in an E. coli isolate (9), the whole-genome sequencing and phylogenetic analysis further revealed a growing trend of ESBL and *mcr-1* coexistence and transmission in human and veterinary medicine (10). Moreover, Feng et al. identified an E. coli isolate with a single plasmid carrying both the *tet*(X6) and *mcr-1* genes, which confers coresistance to both colistin and tigecycline (11). To detect colistin-resistant E. coli mediated by *mcr* genes, rapid and reliable antibiotic susceptibility testing (AST) is essential.

Disk diffusion, broth dilution, and commercially accessible semi-automated systems are the most often utilized AST techniques in clinical practices. Despite being cost-effective and accurate, traditional AST is time-consuming and labor-intensive, with a wait time of approximately 24 h (12). Commercial automated methods like MicroScan WalkAway, Vitek-2, BD PhoenixTM, and SensititreTM are now routinely utilized in clinical practice, reducing AST time from 6 to 16 h and challenging the boundaries of bacterial diagnosis (13). While next-generation sequencing has reduced the cost and quantity of testing known resistance genes, it is also a separate detection of genotype and phenotype (14). Quantitative analysis of antibiotic-responsive RNA responses may quickly distinguish the resistant pathogen strains, independent of resistance mechanisms or genetic background (15). Accordingly, RNA-based transcriptional changes have been applied to evaluate antibiotic susceptibility in a variety of strains including Enterobacteriaceae (16–19). Quantifying changes in RNA signatures following antibiotic treatment is particularly promising for rapid AST.

In the current study, on the basis of bacterium-antibiotic model systems, we developed a quick and accurate RNA-based test for identifying both *mcr-1*-positive and colistin-resistant bacteria. After 60 min of colistin exposure, this rapid AST method was developed based on the significant differences in transcriptome responses between the colistin-susceptible strain (DH5α-pUC19) and the colistin-resistant strain (DH5α-pUC19-*mcr-1*). The candidate RNA markers were verified using quantitative real-time PCR (RT-qPCR) temporal and colistin concentration shifts, and correlated with traditional AST. Following colistin exposure, the potential RNA markers were further validated in other *mcr-1* variants and clinical isolates.

## RESULTS

### Different transcriptome responses between colistin-susceptible and *mcr-1*-mediated colistin-resistant bacteria.

**FIG 1:**
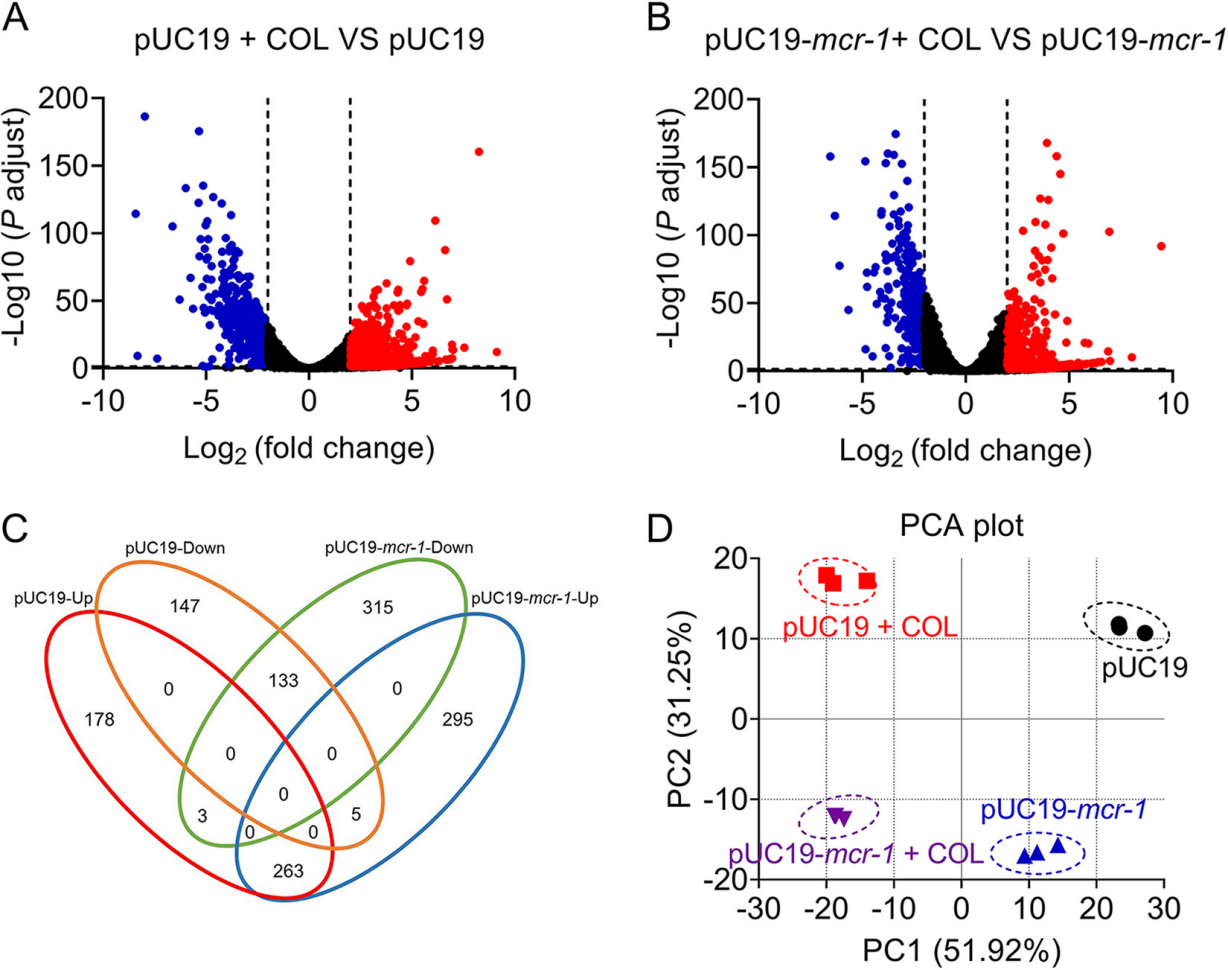
Differential gene expression of *mcr-1*-negative and -positive strains under colistin exposure. (A and B) Volcano illustration of differentially expressed genes from colistin treatment samples of colistin susceptible (DH5α-pUC19) and resistant (DH5α-pUC19-*mcr-1*) strains relative to their control groups. Upregulated genes are indicated by red points (Log_2_FC ≥ 2 and *P < *0.05), and downregulated genes are indicated by blue points (Log_2_FC ≤ −2 and *P < *0.05). (C) Venn diagrams show the number of mRNA biomarkers expression significantly altered by colistin susceptible (DH5α-pUC19) and resistant (DH5α-pUC19-*mcr-1*) after colistin exposure. FDR < 0.05, *P < *0.05 and Log_2_FC ≤ −2 or ≥ 2 (one-way ANOVA). (D) Principal-component analysis (PCA) score plots for transcriptional levels from samples colistin-susceptible (DH5α-pUC19) and -resistant (DH5α-pUC19-*mcr-1*) with or without colistin treatment.

To identify the specific RNA transcripts capable of distinguishing colistin-susceptible and -resistant bacteria, RNA sequencing was used to compare the transcriptional profiles between the reference colistin-susceptible strain (DH5-pUC19) and *mcr-1* positive colistin-resistant strain (DH5-pUC19-*mcr-1*) treated with colistin at a breakpoint concentration for 60 min. Interestingly, we found that colistin-susceptible and -resistant strains displayed different transcriptional responses to 2 μg/mL colistin exposure. As shown in Fig. 1A and B, upon colistin treatment, 562 genes were upregulated and 451 genes were downregulated in DH5-pUC19. By comparison, the DH5-pUC19-*mcr-1* group had 443 upregulated genes and 283 downregulated genes. There were 263 differentially expressed genes (DEGs) in both upregulated groups and 133 DEGs in the downregulated groups (Fig. 1C). Compared with the DH5-pUC19-*mcr-1* group, more DEGs were observed in *mcr-1* negative groups after colistin exposure. Furthermore, the significantly changed genes (Log_2_FC ≥ 2 or ≤ −2, *P ≤ *0.05, ANOVA) were selected for additional confirmation. Principal-component analysis (PCA) results demonstrated a similar change direction between colistin susceptible and *mcr-1* positive groups after colistin exposure, but with a totally different location (Fig. 1D).

### Functional enrichment of DEGs.

**FIG 2:**
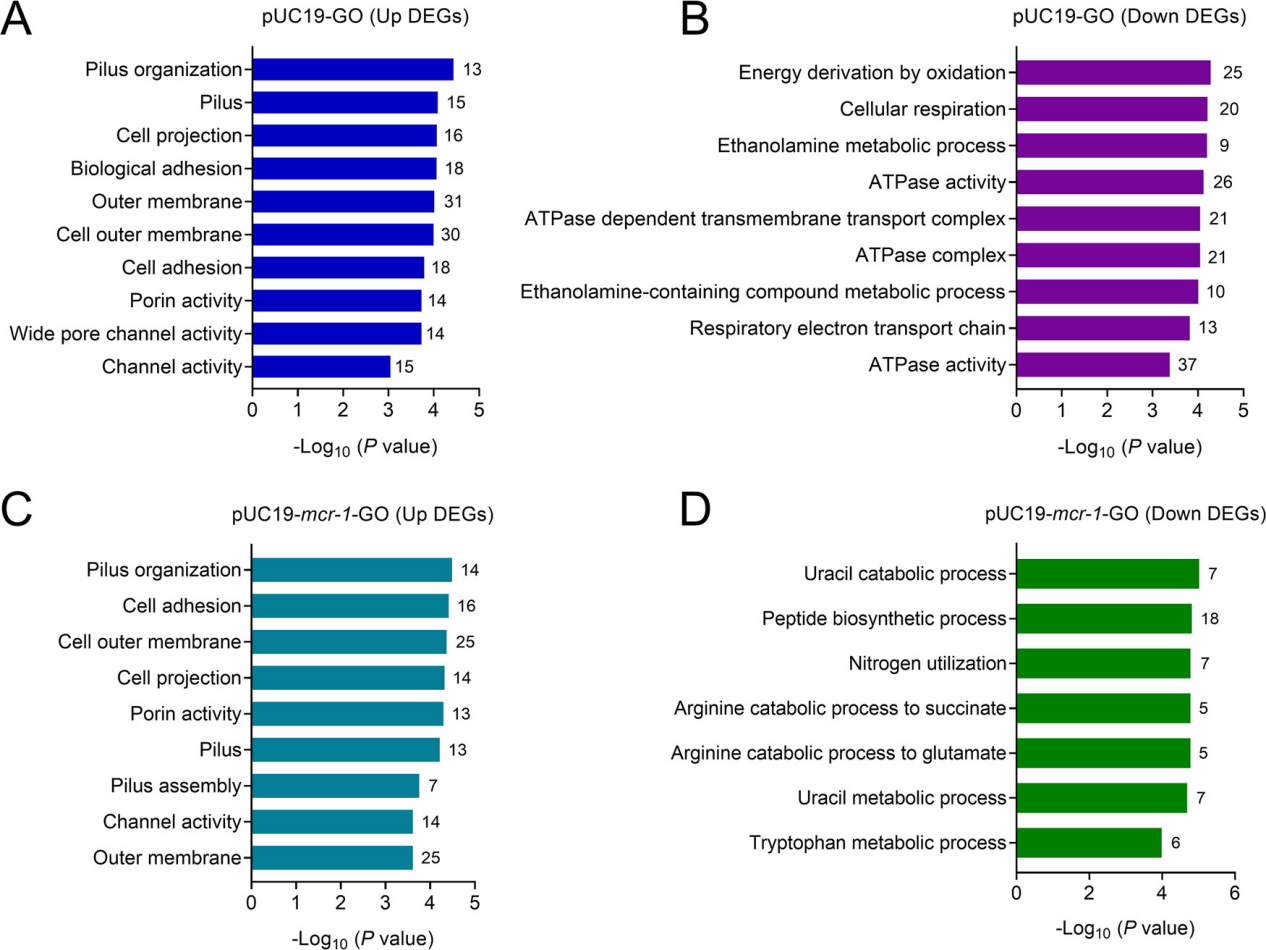
Gene ontology (GO) pathway enrichment of differentially expressed genes after colistin treatment. GO pathway enrichment in colistin-susceptible (DH5α-pUC19) (A and B) and -resistant (DH5α-pUC19-*mcr-1*) strains (C and D) after colistin treatment relative to their control groups. FDR < 0.05, *P < *0.05 and Log_2_FC ≤ −2 or ≥ 2 (one-way ANOVA).

A universal gene ontology (GO) analysis pathway was annotated for functional annotation to further comprehend the functional enrichment of transcriptome results (Fig. 2). The results revealed that the mRNA expression of genes involved in pilus production and adhesion was significantly increased. The antibacterial activity of colistin is dependent on the electrostatic contact between the positively charged colistin and the negatively charged phosphate group of lipid A on LPS located on the bacterial outer membrane. After diffusing through the periplasm from the outer membrane, colistin can intercalate into the inner membrane and produce holes, leading to bacterial lysis. In line with colistin's mechanisms of action, the transcription levels of the outer membrane, porin, and channel activity-related genes were remarkably upregulated. Furthermore, we found that colistin-specific susceptibility genes were enriched in cellular respiration, ATPase activity, ethanolamine metabolic process, and energy derivation by oxidation in DH5α-pUC19 downregulated group (Fig. 2A and B), while genes related to uracil and tryptophan metabolic process and nitrogen utilization were enriched in DH5α-pUC19-*mcr-1* downregulated groups (Fig. 2C and D). These results suggest that colistin-susceptible strains display different changes compared with *mcr-1*-mediated colistin-resistant groups after treatment with colistin at breakpoint concentration (2 μg/mL).

### Selection of candidate RNA biomarkers for fast AST.

**FIG 3:**
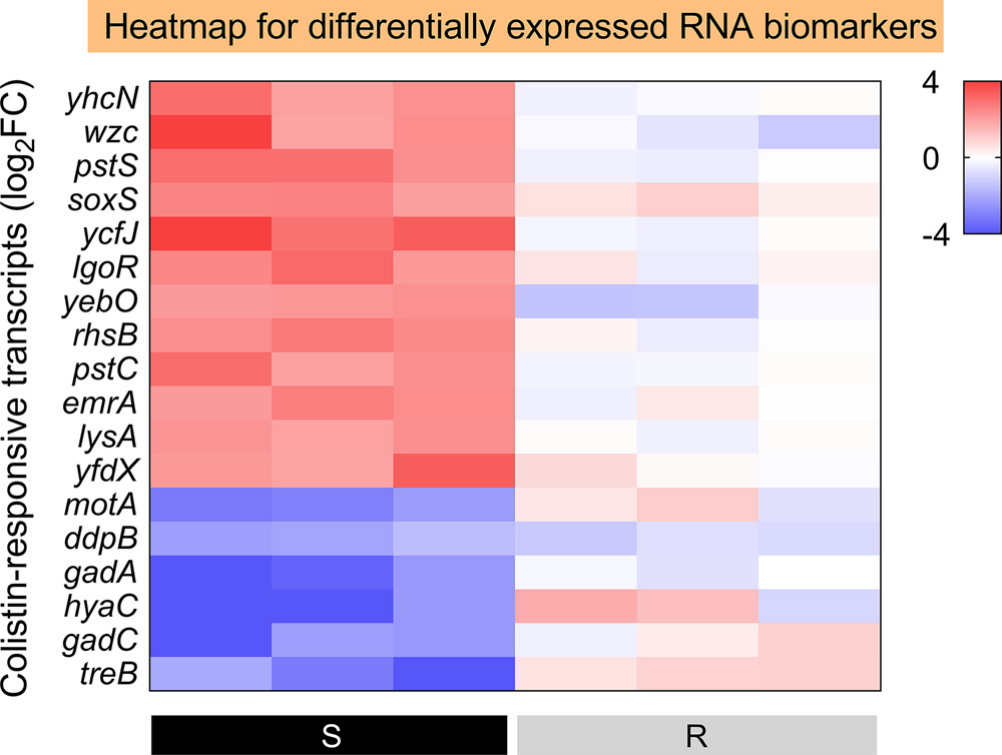
RBAST distinguishes colistin-susceptible and *mcr-1*-conferred colistin-resistant strains. Heatmap of 18 RNA biomarkers in colistin-susceptible and *mcr-1*-mediated colistin-resistant isolate after colistin treatment relative to their control groups. Left black panels represent colistin-susceptible isolates, and right gray panels represent *mcr-1*-mediated colistin-resistant isolates. 16s rRNA was employed as a reference gene.

Genes with a substantial increase or reduction in DH5α-pUC19, but no significant change in DH5α-pUC19-*mcr-1*, were included in the putative colistin-specific susceptibility gene list. A minimum Log_2_FC ≤ −2 or ≥ 2 (*P < *0.05) was required as a significant change in transcriptome profiles and sorted by *P* value. The first-step candidates are shown in Table S3 in the supplemental material. With regard to RT-qPCR-based verification of mRNA biomarkers for a fast molecular AST, three colistin-susceptible and three *mcr-1*-mediated colistin-resistant E. coli isolates with clear backgrounds were utilized. A minimum ΔΔCT value ≤ −2 or ≥ 2 was identified as “significantly differential” in quantitative analysis using RT-qPCR analysis with optimized primers (Table S4). According to Fig. 3 and Fig. S1, 18 of 94 candidate mRNA biomarkers showed significantly differential expression levels between colistin-susceptible and *mcr-1*-mediated groups after colistin treatment. In particular, 12 (*yhcN*, *wzc*, *pstS*, *soxS*, *ycfJ*, *lgoR*, *yebO*, *rhsB*, *pstC*, *emrA*, *lysA*, and *yfdX*) of 18 genes examined were determined as highly upregulated mRNA biomarkers in all three susceptible isolates, but none in the three *mcr-1* positive isolates. Furthermore, only six mRNA biomarkers (*motA*, *ddpB*, *gadA*, *hyaC*, *gadC* and *treB*) were found to be significantly downregulated in colistin-susceptible isolates, whereas no change in the three *mcr-1* positive isolates.

### Colistin concentration shifts in candidate mRNA biomarkers upon colistin treatment.

**FIG 4:**
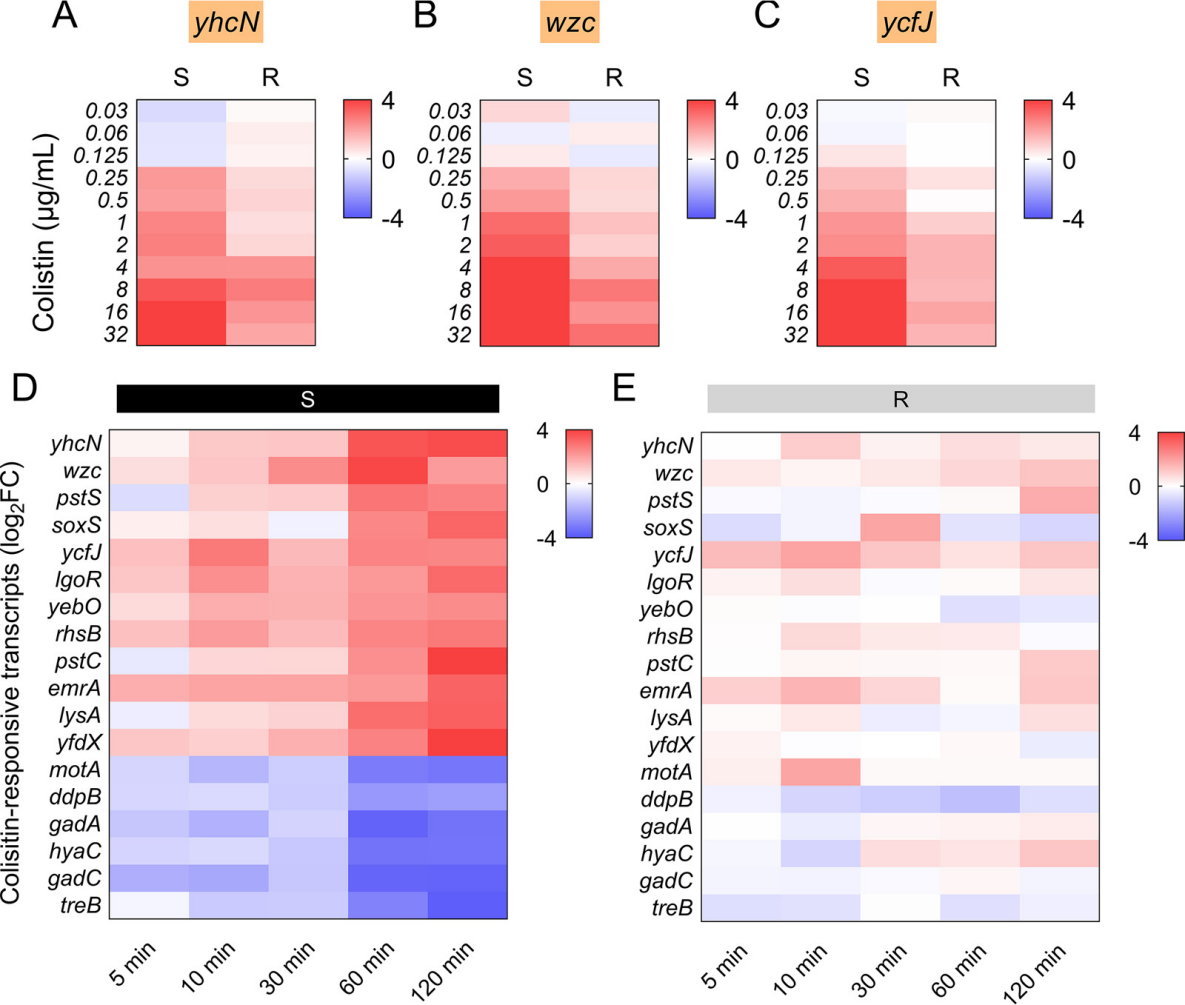
Expression levels of selected RNA biomarkers under different colistin treatment concentrations and times. (A–C) Heatmap of the expression of *yhcN* (A), *wzc* (B), and *rplE* (C) markers in colistin-susceptible and -resistant strains under different colistin concentrations ranging from 0.03 to 32 μg/mL. (D and E) Heatmap of the expression of 18 differentially expressed RNA biomarkers in colistin-susceptible (D) and -resistant (E) strain under different colistin exposure durations. Black panels represent the colistin-susceptible strain and gray panels represent an *mcr-1*-mediated colistin-resistant isolate. 16s rRNA was employed as a reference gene.

In this study, 18 candidate RNA biomarkers were identified between colistin-susceptible and *mcr-1*-mediated colistin-resistant isolates after exposure to 2 μg/mL colistin, and the potential of these mRNA profiles as detection biomarkers to be affected by changes in colistin concentration was further investigated. E. coli ATCC25922 and an *mcr-1*-mediated colistin-resistant clinical E. coli strain were treated with different concentrations of colistin ranging from 0.03125 to 32 μg/mL. After a 60-minute colistin treatment, bacterial RNA was collected and the putative 18 mRNA biomarkers were quantified by RT-qPCR analysis. Three genes in particular, *yhcN*, *wzc*, and *ycfJ*, exhibited a dose-dependent increase in regulation as long as colistin concentrations were high enough (Fig. 4A to C; Fig. S2A to C). Candidate mRNA biomarkers were considerably upregulated in ATCC25922 when the colistin concentration reached 0.25 μg/mL, which corresponded to the MIC value of ATCC25922, but no change was seen in the *mcr-1* positive isolate. When the colistin concentration approached 2 μg/mL, equivalent to the MICs of the *mcr-1* positive isolates, the expression levels of putative mRNA biomarkers in the *mcr-1* positive isolate exhibited a similar response as the susceptible isolate. These findings illustrate that exposure to colistin at breakpoint concentrations may be used to discover distinct mRNA biomarkers that can distinguish colistin-sensitive isolates from *mcr-1*-mediated colistin-resistant bacteria.

### Temporal shifts in candidate mRNA biomarkers upon colistin treatment.

The influence of varied incubation durations on the expression levels of putative mRNA biomarkers was further explored. E. coli ATCC25922 and an *mcr-1*-medicated colistin-resistant clinical E. coli strain were treated with 2 μg/mL colistin for 5 to 120 min. A global shift of 18 potential mRNA biomarkers in ATCC25922 was raised in a relatively short period (10 min), and peaked at around 60 min following colistin treatment, as illustrated in Fig. 4D and E and Fig. S3. However, up to 120 min after colistin administration, there was no significant change in the expression of potential mRNA biomarkers in *mcr-1* positive groups. These findings imply that these putative mRNA biomarkers are responsive to colistin treatment, allowing for quick recognition of *mcr-1*-mediated colistin-resistant E. coli.

### Validation of candidate mRNA biomarkers in *mcr-1* variants.

**FIG 5:**
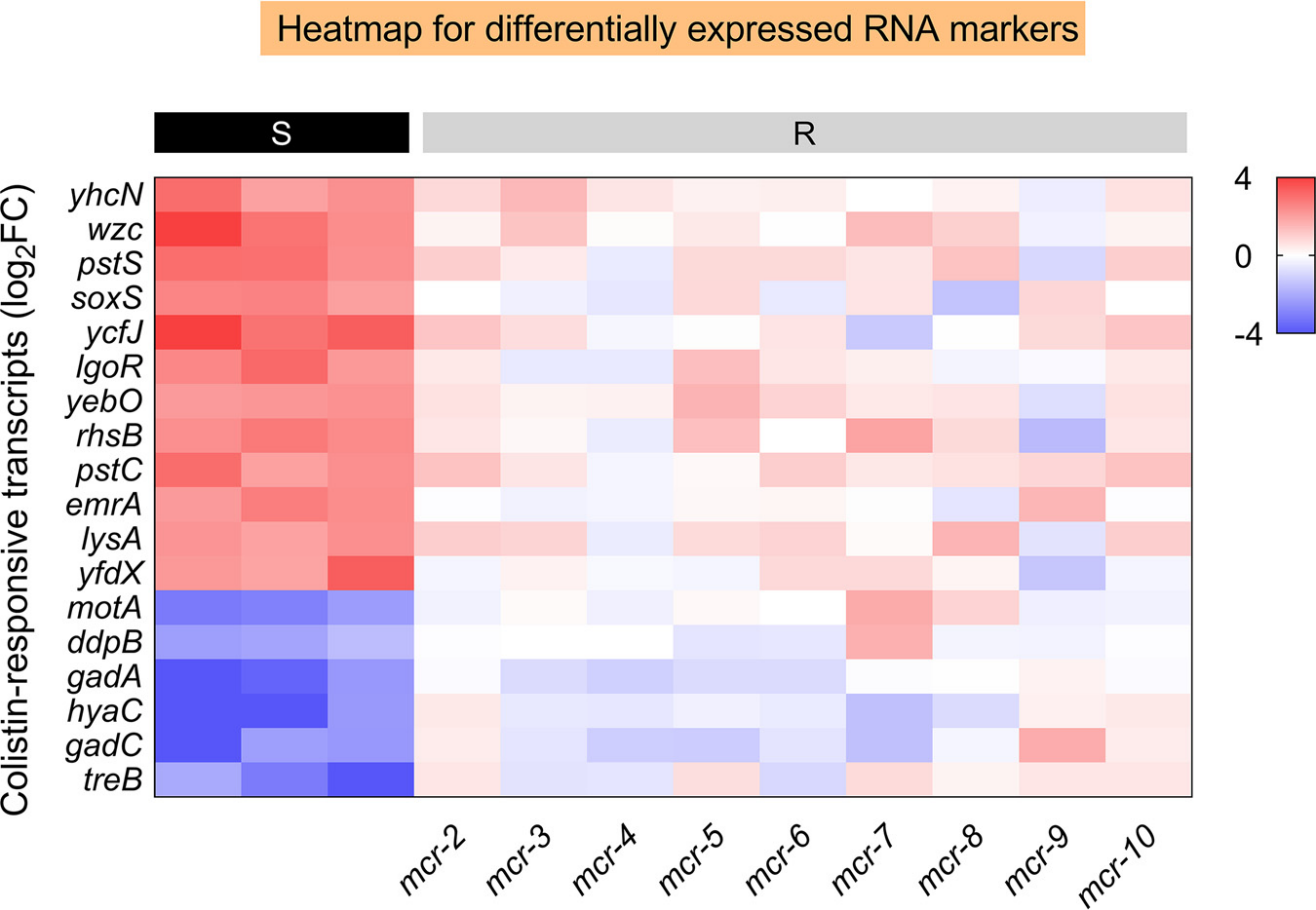
RBAST detects different variants of *mcr-1* using the candidate RNA biomarkers. Heatmap of 18 differentially expressed RNA biomarkers validation across colistin-susceptible and engineered colistin-resistance strains mediated by different variants of *mcr-1* after colistin exposure relative to their control groups. Black panels represent susceptible E. coli, and gray panels represent the construction of different variants of *mcr-1*. 16s rRNA was employed as a reference gene.

The CDS of nine *mcr* variants (*mcr-2*–*mcr-10*) obtained from the NCBI database were cloned into pET23a(+) and transformed into BL21(DE3) to determine if these candidate biomarkers can be utilized to detect colistin-resistant E. coli strains produced by other *mcr* variants. All of the constructs had a low-level colistin resistance phenotype (MIC ≥ 2 μg/mL). After colistin exposure, the expression levels of putative mRNA biomarkers of different *mcr-1* variants were similar to the *mcr-1* positive groups (Fig. 5 and Fig. S4). These findings imply that the potential mRNA biomarkers can be used for the quick molecular AST of colistin-resistant E. coli isolates mediated by *mcr-1* and *mcr* variants.

### Accuracy of RBAST in clinical isolates.

**FIG 6:**
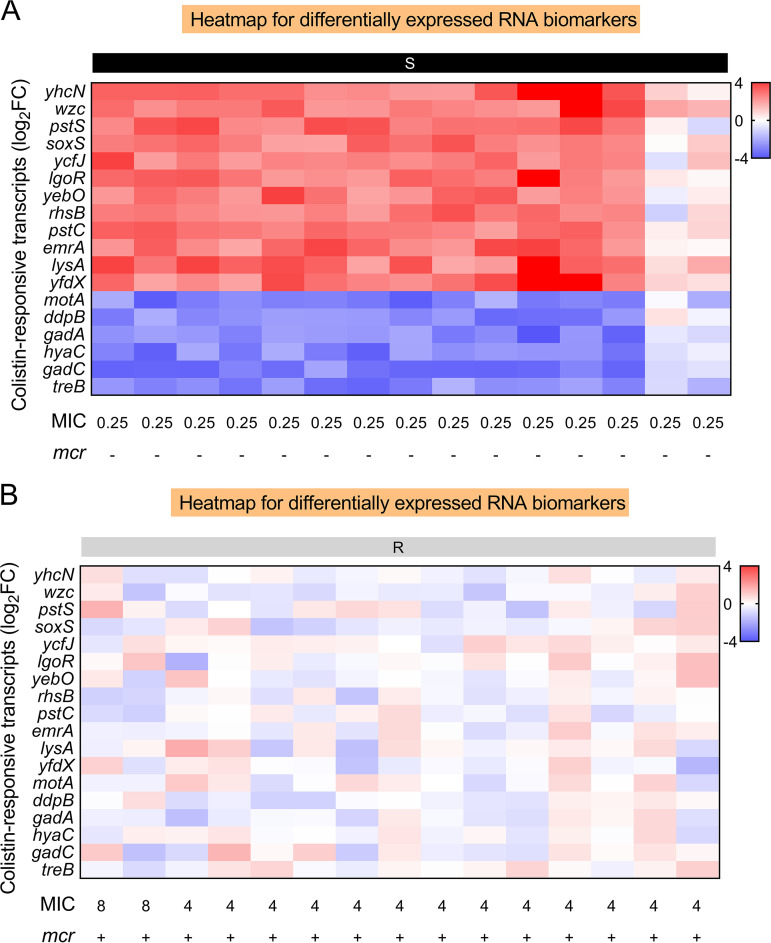
RBAST accurately identifies the susceptibility of E. coli clinical isolates to colistin. Heatmap of 18 selected RNA biomarkers across clinical isolates including colistin-susceptible (A) and *mcr-1*-mediated colistin-resistant E. coli (B) after colistin exposure relative to their untreated groups. Black panels represent colistin-susceptible isolates, and gray panels represent *mcr-1*-medicated colistin-resistant isolates. 16s rRNA was employed as a reference gene.

Thirty clinical E. coli isolates randomly selected were tested for RT-qPCR-based confirmation and MIC correction to further verify the accuracy and possible applicability of RBAST. For higher accuracy, a minimum Log_2_FC ≤ −2 or ≥ 2 (*P < *0.05), and up- or downregulation of at least 16/18 of selected RNA biomarkers were defined as “colistin susceptible” in quantitative analysis using RT-qPCR. On the contrary, at least 16/18 of selected mRNA biomarkers of −2 ≤ ΔΔCT ≤ 2 were needed as no significantly differential regulation and defined as “*mcr* mediated-colistin resistance.” The results of RBAST were compared with traditional MIC tests (Fig. 6 and Fig. S5). According to the RT-qPCR results, 17/30 isolates were defined as “colistin resistance” and 13/30 as ‘’colistin susceptible.” Compared with the MIC results, the RBAST correctly classified 28 of 30 strains (95% categorical agreement), including all 15 colistin susceptible isolates and 13 of 15 resistant isolates, with over 93% accuracy. These results suggest that the RBAST can efficiently distinguish *mcr*-mediated colistin resistance in the clinical situation.

## DISCUSSION

The prevalence and wide spread of *mcr-1* and its variants in humans, animals, and environmental niches calls for more effective AST methods for deploying effective therapeutic regimens. In this research, the transcriptome results of DH5α-pUC19 and DH5α-pUC19-*mcr-1* after treatment with colistin were characterized, and we found that colistin-susceptible and *mcr-1*-mediated colistin-resistant strains displayed different RNA transcripts in some biological functions. Utilizing the differences in mRNA expression between colistin-susceptible and *mcr-1* positive isolates, we constructed a quick and effective AST method called RBAST for assessing colistin susceptibility in bacterial strains.

Traditional AMR detection methods, such as growth-dependent assays, are currently widely used, but most of them require preliminary bacterial isolation, enrichment, and identification steps by culturing in the presence and absence of the relevant antibiotic, which is time-consuming and may delay appropriate antibiotic treatment (20, 21). Several detection approaches have been developed in recent years to improve AST efficiency by shortening the time necessary for isolation, enrichment, or susceptibility determination (22, 23). However, some economic and technical limitations of these methods still limit their clinical translation. For example, high-throughput genotypic detection of *mcr-1* using PCR has high efficiency and sensitivity, but due to a lack of universal primers for each variant, it cannot discover unknown resistance genes (24–26). Additionally, Dekker et al. developed an approach to detect *mcr-1*-containing isolates by characterizing MCR-1 tryptic peptides after protein extraction based on triple quadrupole LC–MS, but this needs a complex sample pretreatment process and intricate analysis of tryptic peptides to MCR-1 (27). Alternative approaches have also been developed, such as nucleic acid-based ASTs, which are performed by utilizing qPCR to determine the number of copies of bacterial chromosomal DNA (28). However, these growth rate-dependent assays are time-consuming, and cannot be corrected by MIC value. On the contrary, a rapid molecular AST based on the candidate RNA biomarkers can yield a rapid and accurate response following short-time antibiotic exposure. For example, a recent report supported that RNA transcripts can be used for rapid detection of ciprofloxacin-resistant Y. pestis and universal antibiotic susceptibility identification of 24 different antibiotics (20, 29). Similarly, RNA biomarkers have been used to aid in the diagnosis of various disorders such as cancer, Parkinson's disease, and other infections (30–32).

More generally, when bacteria are exposed to antibiotics at the breakpoint concentrations, the mRNA transcriptome profiles of antibiotics-susceptible and resistant isolates change dramatically (17, 33, 34). For example, in a previous study of *tet*(X4)-mediated tigecycline-resistant bacteria, we found only 40 upregulated genes in the *tet*(X4)-positive group, compared to 410 in the tigecycline susceptible group, indicating that the transcriptome of the *tet*(X4)-positive group did not respond significantly to tigecycline exposure (35). However, in the current study, once exposed to 2 μg/mL colistin, similar DEGs were sorted out between colistin susceptible and *mcr-1*-mediated colistin-resistant positive groups. Additionally, two groups displayed some similar GO analysis results, including the increased expression of synthesis of pilus, outer membrane, porin, and channel activity-related genes. We suppose that this may be due to the low-level colistin resistance caused by MCR-1, and the treatment with 2 μg/mL colistin poses a considerable burden for *mcr-1* positive groups, leading to more DEGs. Many investigations have consistently demonstrated that the *mcr-1* gene induces low-level resistance in E. coli and K. pneumoniae, with MIC values ranging from 2 to 8 μg/mL (4, 36).

In summary, we developed a quick and comprehensive molecular AST by assessing the fold changes in candidate mRNA biomarkers expression following colistin exposure to distinguish colistin-susceptible from colistin-resistant isolates. The candidate mRNA biomarkers are successfully verified across colistin exposure temporal and concentration shifts in E. coli isolates. The accuracy of colistin susceptibility determination based on the candidate mRNA biomarkers compared with traditional MIC is over 93%. Moreover, the RBAST has been verified and can be extended to examine other pathogens that carry *mcr* variants. Nevertheless, mRNA-based AST can only identify susceptibility categories, not accurate MIC values (20), and requires substantial additional work to yield clinically viable diagnostic protocols.

## MATERIALS AND METHODS

### Strains.

E. coli DH5α was utilized as a reference strain in this study. Clinical colistin-resistant E. coli used in the following experiments were isolated from Père David's Deer (*Elaphurus davidianus* or *milu*) (37), available as Table S1, and preserved in the College of Veterinary Medicine, Yangzhou University, China.

### Plasmids and strains construction.

The standard *mcr-1* gene with its promoter was amplified by PCR using KeyPo Master Mix (Vazyme, China), and cloned into pUC19 using a restriction enzyme site, XbaI, and EcoRI. Other complete coding DNA sequences (CDS) of *mcr* variants were amplified from synthetic gene sequences based on sequences published in NCBI or genomic template and inserted into pET23(a) after T7 promoter using NdeI and BamHI. The primers are shown in Table S2. The produced plasmid named pUC19-*mcr-1* was transformed into DH5α, and other *mcr-1* variants in pET23(a) were transformed into BL21(DE3). E. coli DH5α-pUC19-*mcr-1* and DH5α-pUC19 were engineered as standard colistin-susceptible and -resistant strains.

### Antimicrobial susceptibility testing.

The broth microdilution method was performed to determine the MICs of colistin (CST), aztreonam (ATM), amoxicillin (AMC), ceftiofur (CFF), doxycycline (DOX), enrofloxacin (ENR), florfenicol (FFC), meropenem (MEM), and streptomycin (STR) for all clinical E. coli isolates, and the results were interpreted according to the Clinical and Laboratory Standards Institute (CLSI) guidelines and the European Committee on Antimicrobial Susceptibility Testing (EUCAST) breakpoints. MIC values of more than 2 μg/mL were used to characterize colistin-resistant strains.

### Colistin treatment for sequencing and RNA extraction.

The colistin-resistant strain (DH5-pUC19-*mcr-1*) and the colistin-susceptible strain (DH5-pUC19) were grown at 37°C in LB broth with 100 μg/mL ampicillin to OD_600_ ≈ 1. The incubations were separated into two groups: the treated group received 2 μg/mL colistin incubated at 37°C for 60 min, whereas the control group received no treatment. After stimulation, the supernatants were removed by centrifugation and samples were chilled in liquid nitrogen for 15 min. TRIzol Reagent was used to extract bacterial RNA according to the manufacturer's instructions (InvitroGen, Carlsbad, CA).

### RNA sequencing and data processing.

An RNA-seq transcriptome library with 2 μg of total RNA was performed using Illumina's TruSeqTM RNA sample preparation kit (San Diego, CA). Then, random hexamer primers (Illumina) were used to synthesize double-stranded cDNA using a SuperScript double-stranded cDNA synthesis kit (Illumina). The library was sequenced by the Illumina HiSeq × 10 (2 × 150 bp read length) after being quantified by TBS380 and processed by Illumina GA Pipeline (version 1.6), yielding 150 bp paired-end reads. The reads were aligned to the E. coli K12 strain (NCBI reference sequence: NC_000913.3). XLSTAT software (2015 version, Addinsoft) was used for the principal-component analysis (PCA).

### Validation of selected RNA biomarker expressions.

The same protocols provided in 2.4 were performed for quantitative real-time PCR (RT-qPCR) validation. MiPure Cell/Tissue miRNA Kit (Vazyme) was used to collect and extract samples according to the manufacturer's instructions. HiScript III RT SuperMix for qPCR (+gDNA wiper) (Vazyme) was used for validation of RNA biomarker expressions. Primers shown in Table S4 were designed to validate candidate genes using Primer Premier 5.1. A relative quantitative method was applied to calculate the fold changes (log_2_FC=-ΔΔCT) of mRNA expression relative to the reference genes (16S rRNA).

### Data availability.

RNA-sequencing data have been deposited in the National Center for Biotechnology Information (NCBI) Sequence Read Archive (SRA) database (PRJNA830332).
