# Supplementary material for: Rapid Detection of MCR-Mediated Colistin Resistance in Escherichia coli
Source: Microbiol Spectr. 2022 May 26;10(3):e00920-22. doi: 10.1128/spectrum.00920-22 (PMC9241874; doi:10.1128/spectrum.00920-22)
Supplement: SUPPLEMENTAL FILE 1 — Supplemental material. Download spectrum.00920-22-s001.pdf, PDF file, 0.4 MB [file spectrum.00920-22-s001.pdf]

Supplementary Figures

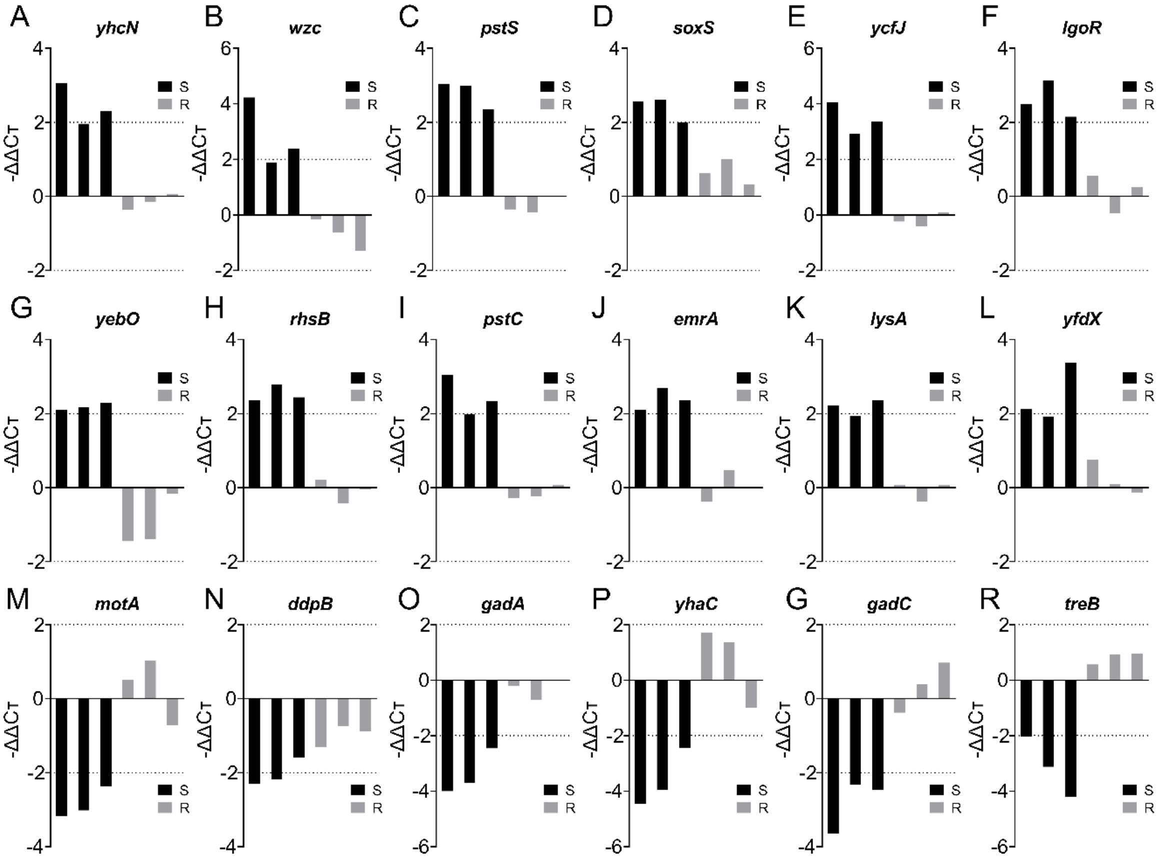

Supplementary figure 1 RBAST distinguishes colistin-susceptible and *mcr-1*-medicated colistin-resistant isolates.

Quantitative real-time PCR of 18 colistin sensitive mRNA biomarkers across three susceptible and three *mcr-1*-medicated colistin-resistant isolates after colistin exposure relative to their control. Black bars indicate susceptible isolates and grey bars indicate resistant isolates. 16s rRNA was used as a reference gene.

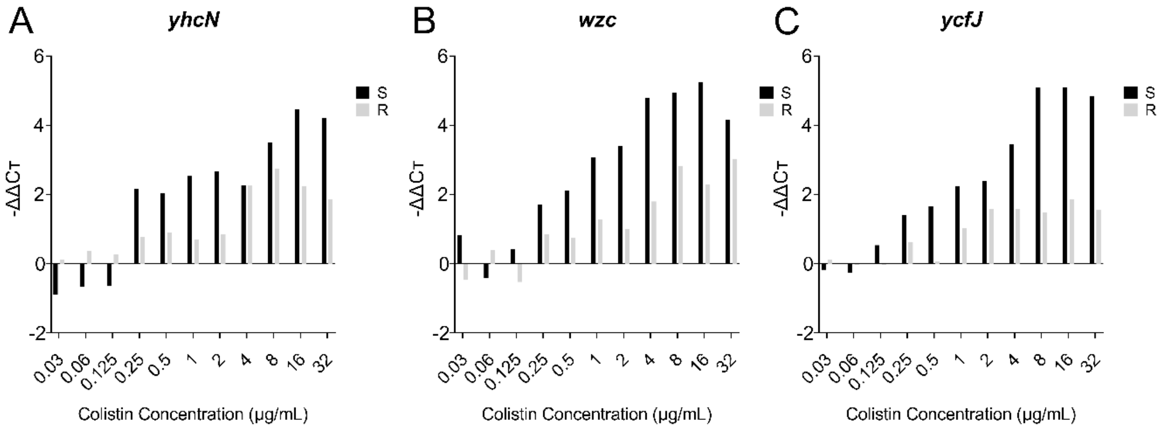

Supplementary figure 2 Expression of candidate mRNA biomarkers upon different colistin exposure concentrations.

Histogram of *yhcN* **(A)**, *wzc* **(B)** and *ycfJ* **(C)** biomarkers demonstrated the most sensitive susceptibility information across the MIC range of colistin. Black bars indicate susceptible *E. coli* ATCC25922 and grey bars indicate *mcr-1*-mediated colistin-resistant isolates. 16s rRNA was used as a reference gene.

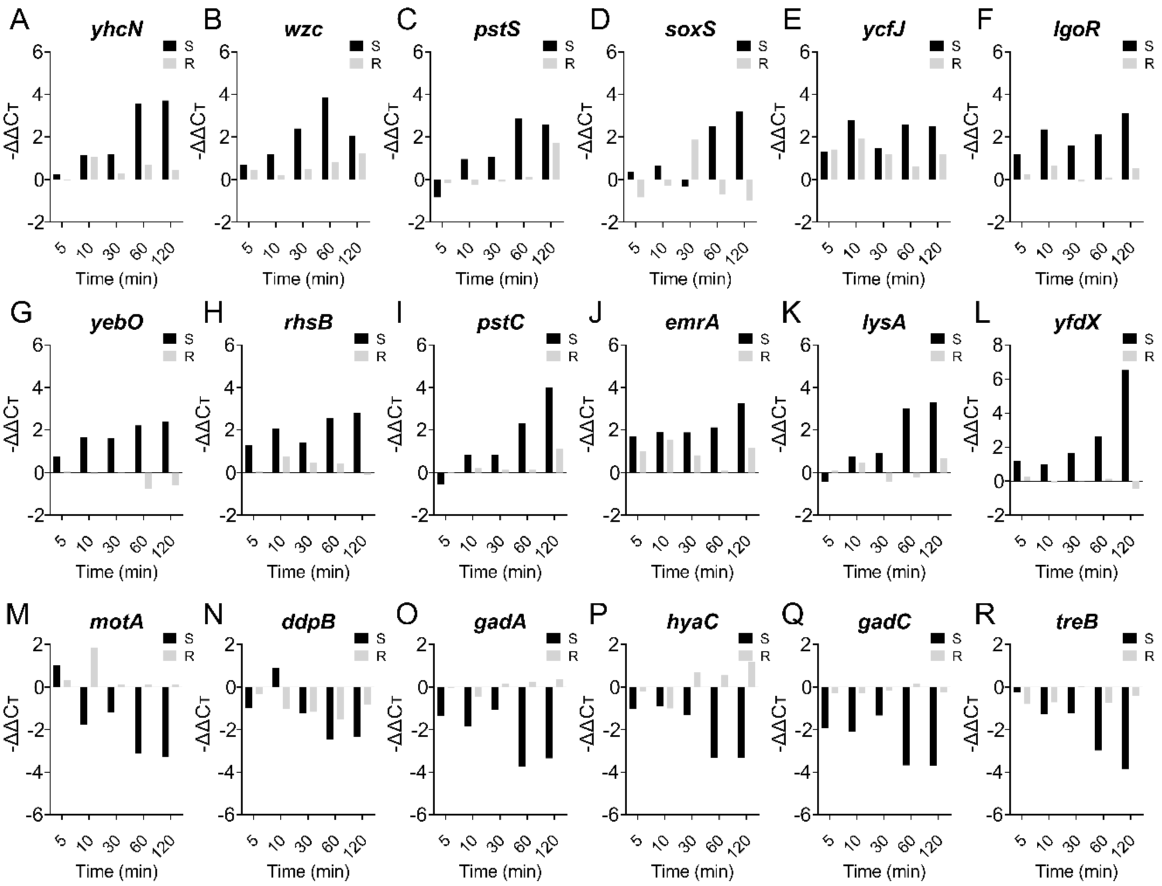

Supplementary figure 3 Expression of candidate mRNA biomarkers upon different colistin exposure times.

Histogram of 18 differentially expressed mRNA biomarkers across exposure duration of colistin. Black bars indicate susceptible *E. coli* ATCC25922 and grey bars indicate *mcr-1*-medicated colistin-resistant isolates. 16s rRNA was used as a reference gene.

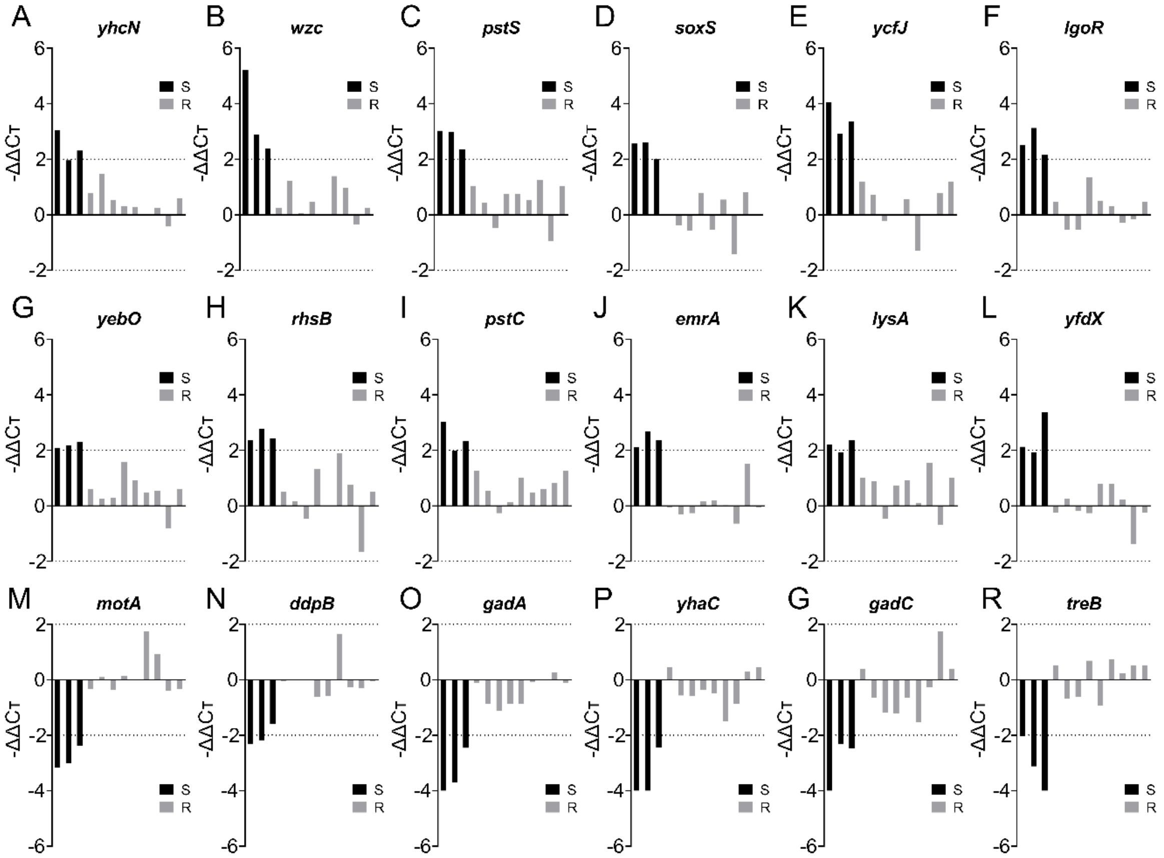

Supplementary figure 4 RBAST detects different *tet*(X) variants using the selected RNA biomarkers.

Histogram of 18 differentially expressed RNA markers validation across susceptible and construction of different variants of *mcr-1* after colistin exposure relative to their control. Black bars indicate susceptible *E. coli* and grey bars indicate *mcr* variant-medicated colistin-resistant isolates. 16s rRNA was used as a reference gene.

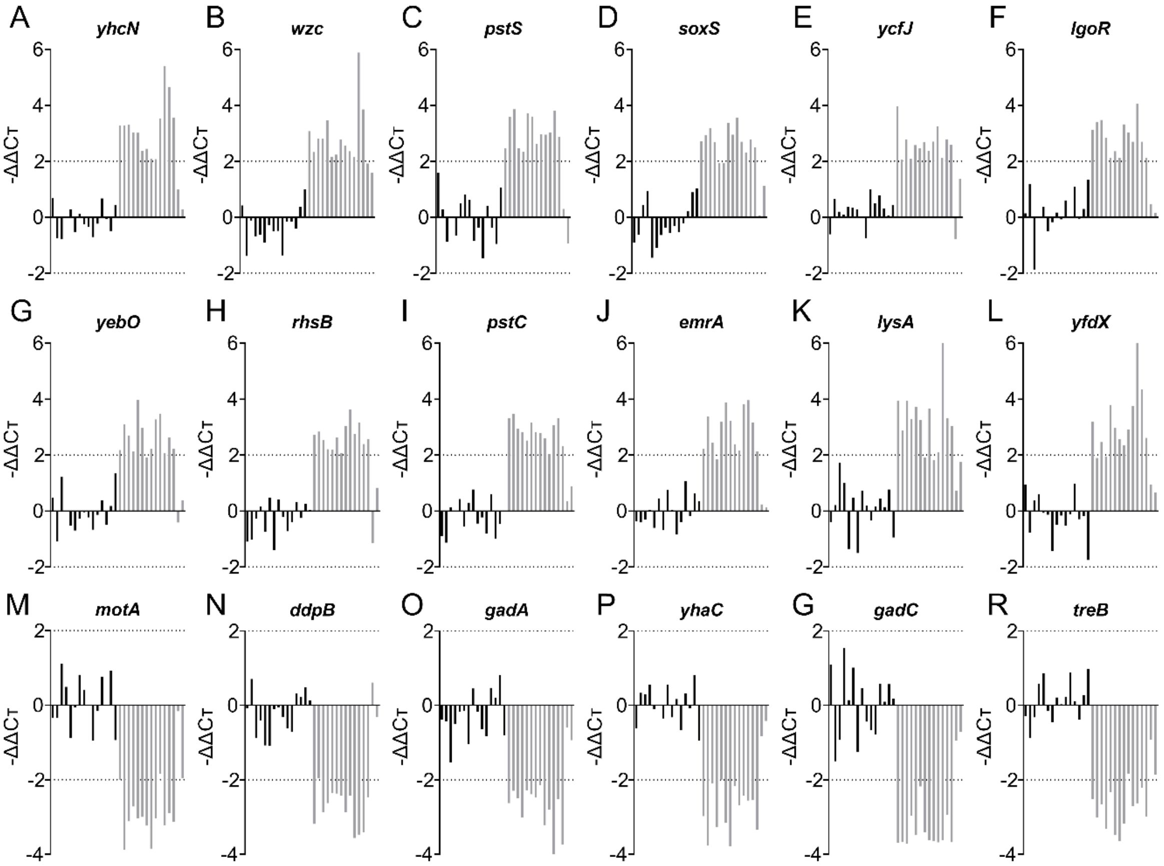

Supplementary figure 5 RBAST accurately classifies *E. coli* isolates.

Histogram of 18 colistin sensitive mRNA biomarkers across clinical isolates susceptible and resistant *E. coli* after colistin exposure relative to their control. Black bars indicate susceptible isolates and grey bars indicate *mcr*-medicated colistin-resistant isolates. 16s rRNA was used as a reference gene.
